# T1DMicro: A Clinical Risk Calculator for Type 1 Diabetes Related Microvascular Complications

**DOI:** 10.3390/ijerph182111094

**Published:** 2021-10-21

**Authors:** Paul Minh Huy Tran, Eileen Kim, Lynn Kim Hoang Tran, Bin Satter Khaled, Diane Hopkins, Melissa Gardiner, Jennifer Bryant, Risa Bernard, John Morgan, Bruce Bode, John Chip Reed, Jin-Xiong She, Sharad Purohit

**Affiliations:** 1 Center for Biotechnology and Genomic Medicine, Augusta University, 1120, 15th Str., Augusta, GA 30912, USA; ptran@augusta.edu (P.M.H.T.); eikim@augusta.edu (E.K.); lytran@augusta.edu (L.K.H.T.); fbinsatter@augusta.edu (B.S.K.); dhopkins@augusta.edu (D.H.); mgardiner@augusta.edu (M.G.); jbryant@augusta.edu (J.B.); ribernard@augusta.edu (R.B.); jshe@augusta.edu (J.-X.S.); 2 Department of Neurology, Medical College of Georgia, Augusta University, 1120, 15th Str., Augusta, GA 30912, USA; jmorgan@augusta.edu; 3 Atlanta Diabetes Associates, Atlanta, GA 30318, USA; BruceBodeMD@atlantadiabetes.com; 4 Southeastern Endocrine and Diabetes, Atlanta, GA 30076, USA; arosenbaum@seedreed.com; 5 Department of Obstetrics and Gynecology, Augusta University, 1120, 15th Str., Augusta, GA 30912, USA; 6 Department of Undergraduate Health Professionals, Augusta University, 1120, 15th Str., Augusta, GA 30912, USA

**Keywords:** type 1 diabetes, complications, peripheral and autonomic neuropathy, retinopathy, nephropathy, risk prediction, clinical calculator

## Abstract

Development of complications in type 1 diabetes patients can be reduced by modifying risk factors. We used a cross-sectional cohort of 1646 patients diagnosed with type 1 diabetes (T1D) to develop a clinical risk score for diabetic peripheral neuropathy (DPN), autonomic neuropathy (AN), retinopathy (DR), and nephropathy (DN). Of these patients, 199 (12.1%) had DPN, 63 (3.8%) had AN, 244 (14.9%) had DR, and 88 (5.4%) had DN. We selected five variables to include in each of the four microvascular complications risk models: age, age of T1D diagnosis, duration of T1D, and average systolic blood pressure and HbA1C over the last three clinic visits. These variables were selected for their strong evidence of association with diabetic complications in the literature and because they are modifiable risk factors. We found the optimism-corrected R2 and Harrell’s C statistic were 0.39 and 0.87 for DPN, 0.24 and 0.86 for AN, 0.49 and 0.91 for DR, and 0.22 and 0.83 for DN, respectively. This tool was built to help inform patients of their current risk of microvascular complications and to motivate patients to control their HbA1c and systolic blood pressure in order to reduce their risk of these complications.

## 1. Introduction

The hyperglycemic state present in type 1 diabetes is associated with both micro- and macrovascular complications [1,2]. Microvascular damage leads to neuropathy, retinopathy, and nephropathy, which are each associated with clinical sequelae. Diabetic peripheral neuropathy (DPN) can lead to poor wound healing, diabetic ulcers, and eventually, amputation [3]. Autonomic neuropathy (AN) can present with cardiac abnormalities, gastroparesis, or erectile dysfunction [4]. Diabetic retinopathy (DR) can lead to blindness [5]. Diabetic nephropathy (DN) can progress to end-stage renal disease, requiring dialysis or renal transplantation [6].

Microvascular complications are major predictors of macrovascular complications, such as myocardial infarctions and cerebrovascular accidents, which are the leading cause of death in the USA [7]. The most effective method to reduce morbidity and mortality in diabetic patients is minimizing the risk of macrovascular complications. This involves (1) identifying modifiable risk factors for developing microvascular complications, (2) motivating patients to reduce their personal risks, and (3) providing patients with tools to achieve risk reduction.

One of the most important modifiable risk factors is glycemic control. Multiple randomized control trials have demonstrated that tight glycemic control is associated with decreased risk of micro- and macrovascular complications [8,9]. Other factors have been associated with risk of microvascular complications in cross-sectional and longitudinal studies, including sex [10], onset of type 1 diabetes during puberty [11], glycemic variability [12], quality of life during adolescence [13], and hypertension [14].

Once modifiable risk factors are identified, clinicians can educate patients on how to reduce these risk factors through lifestyle changes and medical management. Informing patients of their personal risks for developing diabetic complications helps patients set a realistic understanding of these risks and allows them to monitor how their lifestyle and/or medication changes have reduced their risks of diabetic complications [15]. Particularly for patients who already suffer from a lifelong, time-consuming, and usually expensive disease, risk scores can help prioritize their future-problem mitigation plan.

Risk scores have been reported in the past for complications of type 1 [16,17,18,19] and type 2 diabetes [20]. Kazemi et al. published a support vector machine model using 13 clinical variables to predict DPN severity with an accuracy of 76% [17]. Lagani et al. used an accelerated failure model on the Diabetes Control and Complications Trial (DCCT) data to predict time to DPN onset using five variables—HbA1C, albumin, age, degree of retinopathy, and duration of postpubescent diabetes—with a concordance index of 0.74 on a test dataset [18]. They similarly used an accelerated failure model to predict time to retinopathy using five variables—HbA1c, marital status, degree of retinopathy, postpubescent diabetes duration, and body mass index—with a concordance level of 0.72 on a test data set. Their random survival forest model for time to microalbuminuria based on six variables—HbA1c, marital status, urine albumin value, insulin regime, degree of retinopathy, postpubescent diabetes duration, and weight—had a concordance level of 0.82 on a test data set. DCCT modeled DPN risk using a generalized estimating equation with the variables mean HbA1c, age, height, duration of T1D, presence of DR, urinary albumin excretion rate, mean heart rate, and use of beta blocker. Braffett et al. also used a generalized estimating equation to model cardiovascular AN risk with the variables age, urinary albumin excretion rate, HbA1c, duration of T1D, mean pulse, beta blocker use, systolic blood pressure (SBP), presence of diabetic retinopathy, macular edema, estimated glomerular filtration rate (eGFR) less than 60, and cigarette smoking status [19]. A risk score was developed for blindness and limb amputation in individuals with type 1 or type 2 diabetes based on cox proportional hazards models [20].

These risk scores have not been implemented in clinics due to several factors. First, these scores use complex statistical approaches that are not easily accessible for patients and clinicians to use for risk calculations. Second, the scores for each complication use different clinical variables, making it more difficult for patients to collect all of the data necessary for computing clinical risk for each complication. Third, these scores do not show patients how changing their modifiable risk factors would change their risk of developing diabetic complications. Scores should be easily accessible and easy to use so that patients can use them to monitor their progress [15]. Learning this risk may motivate patients to progress on the Prochaska and DiClemente stages of change [21].

With the increasing incidence and survival of patients with T1D [22,23,24], these complications are becoming more important to study. We used a cross-sectional study, Phenome and Genome of Diabetes Autoimmunity (PAGODA), to develop a clinical risk score for DPN, AN, DR, and DN in patients with T1D.

## 2. Materials and Methods

### 2.1. Study Population

Individuals diagnosed with T1D who attended the Augusta University (AU) Medical Center and/or endocrinology clinics in Augusta and Atlanta areas of Georgia between 2002 and 2010 were recruited into the Phenome and Genome of Diabetes Autoimmunity (PAGODA) study [25,26]. For consented patients, demographic and clinical variables, including age, sex, date of T1D diagnosis, medical diagnoses, blood pressure, and laboratory measurements, were extracted for the last three clinic visits (Table 1). Diagnoses of DPN, AN, and DR were extracted from patients’ electronic health records. DN was diagnosed by the physician/endocrinologist based on the last three microalbumin/creatinine ratio (MACR) values. We used MACR > 30 for the diagnosis of DN. The vast majority of subjects were diagnosed with DPN based on a neurological history and exam by the treating endocrinologist. Many, but not all, had further evaluations and confirmation of DPN by a neurologist. DR was diagnosed by the yearly screening fundoscopic exam, and patients with concerning findings were referred to an ophthalmologist for diagnosis and treatment. The research was carried out according to The Code of Ethics of the World Medical Association (Declaration of Helsinki, 1997). All study participants gave written informed consent. The study was reviewed and approved by the institutional review board at AU.

### 2.2. Statistical Analysis

Continuous data were presented as median and interquartile range, differences between the groups were tested by Kruskall–Wallis test. Chi-square test was used for count data. The potential differences between T1D patients with and without each complication (DPN, AN, DR, and DN) were initially examined using univariate logistic regression. We selected five variables that showed consistent associations with diabetic complications across multiple studies and were modifiable through lifestyle and medication management: age, age at T1D diagnosis, duration of T1D, and average HbA1C and SBP over the last three clinic visits. These five variables were used to construct four multiple logistic regression models. Each model produced a clinical risk score for each diabetic complication in a microvascular-naive T1D patient.

We determined the linearity of the relationship between each continuous variable and the microvascular complication using spiked histograms for visual analysis and analysis of variance of restricted cubic spline fits of the data for the statistical test of linearity. We used the spiked histograms to pre-specify the number of knots used for restricted cubic splines appropriate for each variable. The knots are placed at equal intervals across the distribution of the variables. These variables were all modeled with restricted cubic splines with 3, 5, and 4 knots, respectively. For the DR model, duration of T1D was also modeled with a restricted cubic spline with 3 knots. Calibration plots and validation were performed using the “calibrate” and “validate” functions, respectively, in the “rms” package [27] with 500 iterations of bootstrapping [28].

All *p*-values were two-tailed, and a *p* < 0.05 was considered statistically significant. All statistical analyses were performed using the R language and environment for statistical computing (R version 3.6.1; R Foundation for Statistical Computing; www.r-project.org, accessed on 14 October 2021). All data and code used to generate models, plots, and the website are available at https://github.com/pmtran5884/T1D_Complications (accessed on 14 October 2021).

## 3. Results

### 3.1. Rates of Diabetic Complications

We consented a cross-sectional cohort of 1647 T1D patients. All patients were Caucasian, their clinical and demographic variables are listed in Table 1. The 1647 T1D patients were further divided into two groups, subjects who do not have any complications (T1D, *n* = 1026) and T1D subjects with any complications (T1D_wComp, *n* = 621). Subjects with complications were older (48.2 vs. 16.2 years, *p* < 0.0001) and had T1D for longer duration (Table 1). Of the 621 subjects with complications, 199 (32.0%) were diagnosed with diabetic peripheral neuropathy (DPN), 63 (10.1%) were diagnosed with autonomic neuropathy (AN), 244 (39.3%) were diagnosed with diabetic retinopathy (DR), and 88 (14.2%) were diagnosed with diabetic nephropathy (DN). Of these, 25 (4%) had diabetic foot ulcers and 17 (2.7%) had limb amputations. Of the patients with DR, 167 (26.9%) have had photocoagulation and 42 (6.8%) were blind (Table 1). There were significant differences in physiological variables such as systolic and diastolic blood pressure, serum levels of creatinine, cholesterol, and LDL (Table 1). There was no significant differences in the HbA1c levels, suggesting good blood glucose controls among the T1D and T1D with complications groups. Percentage of current smokers was higher in the complication group.

### 3.2. Individual Risk Factors Associated with Diabetic Complications

Univariate logistic regression analyses included several statistically significant results (Table 2 and Supplementary Table S1). All four complications were associated with age, age at T1D diagnosis, and duration of T1D, as well as average SBP, DBP, and blood urea nitrogen, dyslipidemia, and a macro-vascular condition (coronary artery disease, myocardial infarction, or cerebrovascular accident).

In lipid panel, no significant associations were observed for LDL and triglycerides. Total cholesterol was associated with DR, and HDL was found to be associated with DPN, AN, and DR (Table 2 and Supplementary Table S1).

Interestingly, smoking status was not associated with any diabetic microvascular complications despite well-established associations between smoking and vascular disease. History of transient ischemic attack and microalbuminuria was associated with DPN, DR, and DN, but not AN; AN had the fewest significant associations with the patient variables in our analyses. It was also interesting that HbA1C was not found to be significantly associated with any of the four complications (average, maximum, or most recent) despite being the mainstay marker of diabetes severity.

### 3.3. Multivariate Predictive Model of Complications

**Figure 1:**
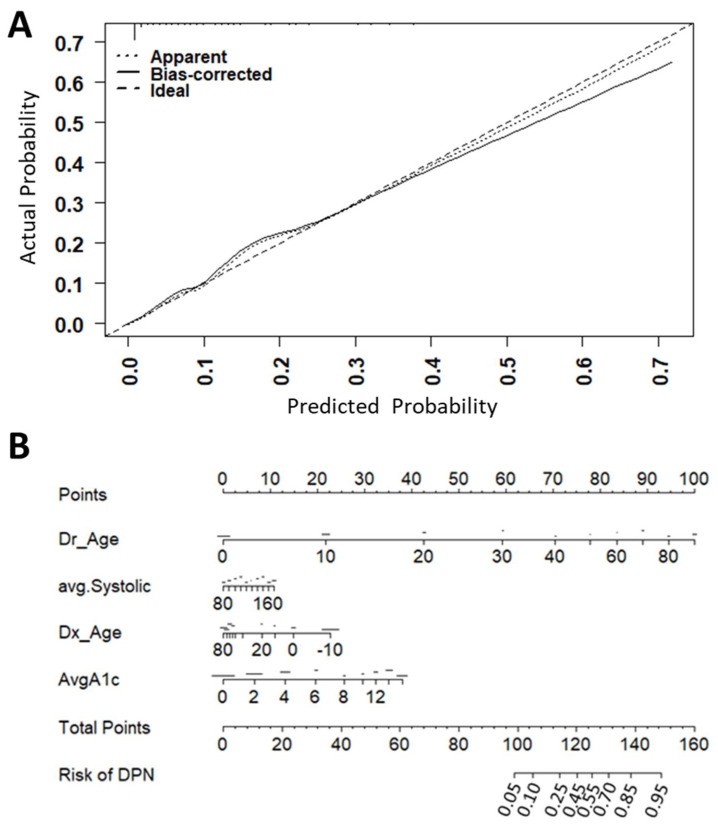
Calibration plot (**A**) and nomogram (**B**) for DPN in a multivariate logistic regression model. Calibration plots and nomograms for AN (C and D), DR (E and F), and DN (G and H) are presented in Supplementary Figure S1. We applied the four microvascular complication models to the PAGODA dataset with 500 iterations of bootstrapping to generate the calibration plot showing the actual probability of complication in the y-axis and the predicted probability of complication based on the models in the x-axis. A bias correction was applied by calculating the difference in probability between the bootstrap iterations and the model prediction with the full dataset. The nomogram was generated again based on the beta coefficients from the four logistic microvascular complication models.

We developed multivariate models to determine the risks of a microvascular complication-naïve T1D patient for developing DPN, AN, DR, and DN based on five variables: patient age, duration of T1D, age at diagnosis of T1D, systolic blood pressure, and HbA1c. Spiked histograms demonstrated a nonlinear relationship between current age, age at diagnosis of T1D, and average HbA1c over three clinic visits and the four diabetic complications (Figure 1).

In order to decrease the model complexity, we reasoned that variability accounted for by duration of T1D may be explained by current age and age of T1D onset. Thus, we compared models with and without this variable. The models without T1D duration were comparable to the models with this variable based on the likelihood ratio test for DPN (*p* = 0.28) and AN (*p* = 0.28), but performed worse for DR (*p* = 3 × 10^−4^) and DN (*p* = 0.04). We compared the calibration plots for each pair of models and found the mean squared error of the bias-adjusted curves were similar for the DPN (2.3 × 10^−4^ with T1D duration, 2.2 × 10^−4^ without T1D duration), AN (4 × 10^−5^ and 6 × 10^−5^, respectively), and DN (8 × 10^−5^ and 2.4 × 10^−4^, respectively). We found the removal of the duration of T1D term gave an acceptable trade-off between model predictions and model interpretability for the DPN, AN, and DN models. The DR model retained the duration of type I diabetes term.

The models were validated using 500 iterations of bootstrapping with replacement. We found the optimism-corrected R^2^ and Harrell’s C statistics were 0.39 and 0.87 for DPN, 0.24 and 0.86 for AN, 0.49 and 0.91 for DR, and 0.22 and 0.83 for DN. The calibration plot for each final model was generated from 500 iterations of bootstrapping with replacement and is presented in Figure 1 and Supplementary Figure S1. Calibration plots demonstrated that all models tend to slightly overfit in the higher risk probability end of the models, and the models for AN and DN overfit more than the DPN and DR models do (Figure 1 and Supplementary Figure S1).

For both the DPN and AN models, the variables in order from most to least contributory were age, age at T1D diagnosis, average HbA1c, and average SBP. For the DR model, the variables that contributed to the risk most were age, average SBP, duration of T1D, age of T1D diagnosis, and average HbA1c. For the DN model, age, age of T1D diagnosis, average SBP, and average HbA1c contributed most to the risk. The contribution of each variable to the risk of each complication is shown as nomograms in Figure 1 and Supplementary Figure S1.

### 3.4. Web Interface to Predict Individual Risk of Diabetic Microvascular Complications

**Figure 2:**
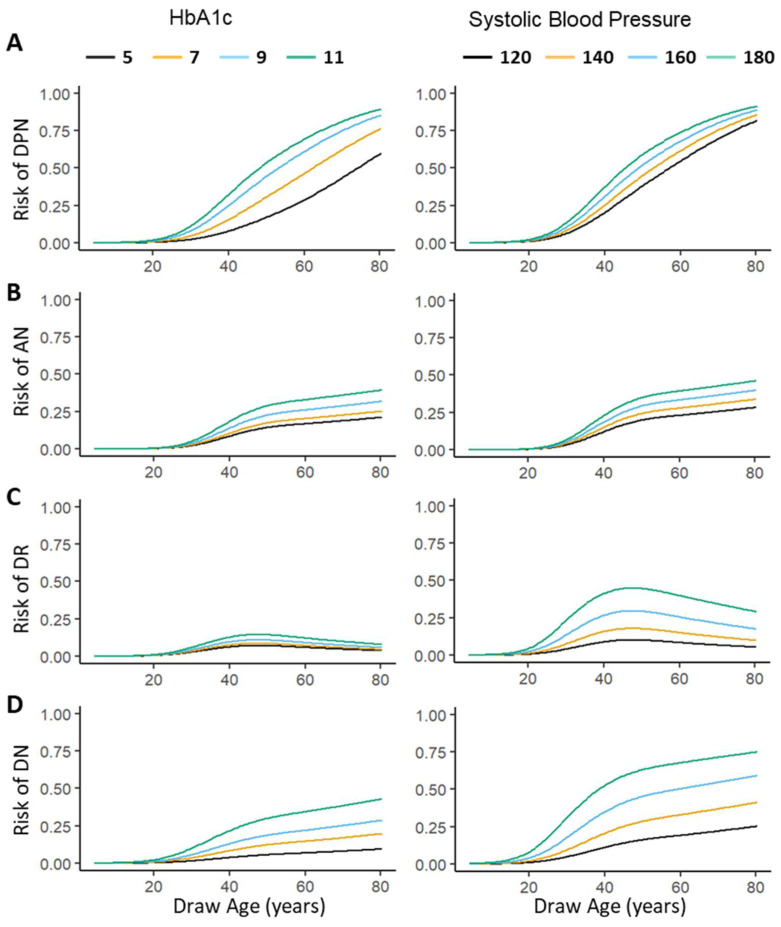
Predicted risk of DPN (**A**), AN (**B**), DR (**C**), and DN (**D**) with increasing HbA1c (**left**) and systolic blood pressure (**right**). We applied the four logistic microvascular complication models to simulated data by fixing the mean and standard deviation of the simulated data to that of the PAGODA population and varying the patient age at day of sample and our variables of interest, HbA1c and systolic blood pressure. We tested HbA1c at 5, 7, 9, and 11. We tested systolic blood pressure at 120, 140, 160, and 180. The gray zones show the 95% confidence interval of the predicted risk.

To facilitate the use of our risk models by clinicians and patients, we created a web interface (https://ptran25.shinyapps.io/Diabetic_Peripheral_Neuropathy_Risk accessed on 14 October 2021) for individuals to estimate their specific risks of diabetic microvascular complications. This interface was created to help inform patients of their personal risks of complications, motivate them to reduce their complication risk by reducing their SBP and HbA1C, and track their progress. In our models, changes in SBP and/or HbA1c levels were associated with noticeable changes in probability of having a microvascular complication (Figure 2). According to the International Society of Hypertension guidelines [29,30], the target SBP for hypertensive patients is less than 140 mmHg. Our algorithm provides an additional risk estimate for patients whose SBP is greater than 140 mmHg had their SBP been 20 mmHg lower. According to the American Diabetes Association, International Society for Pediatric and Adolescent Diabetes, and Canadian Diabetes Association guidelines [31,32,33], the target HbA1C for diabetic patients is less than 7%. For patients whose HbA1C is greater than 7%, our algorithm also provides their risks had their HbA1C been 2% lower than that entered. These additional risk scores for patients who have not met recommended SBP and HbA1C goals are intended to provide patients with information about what their risks would be with improved blood pressure and blood glucose control.

## 4. Discussion

While the DCCT trial and numerous other studies have shown the importance of HbA1C levels in the risk of diabetic complications [8,9], our cross-sectional cohort had no association between any of the four diabetic complications and average HbA1c, maximum HbA1c, standard deviation of HbA1c, or most recent HbA1c on univariate analysis (Table 2). However, average HbA1c was a significant contributor in the multivariate logistic regression models (Figure 1), suggesting that HbA1c is important in the context of age and age at T1D diagnosis. Additionally, while the DCCT clinical trial compared glucose control through HbA1c in separate arms, our cross-sectional non-interventional cohort has lower HbA1c values on average and the HbA1c values were more closely distributed (Table 1). Additionally, we did not identify an association between smoking status and microvascular complications. It is possible that our patient population under-reported smoking or that the lack of pack years in our analysis led to the lack of statistical significance.

Most of the results from the univariate risk models agree with previous reports of risk factors for complications (Table 2). Similar to previous reports, we found a significant association between blood pressure, hypertension, and the four diabetic complications [14]. We found that dyslipidemia was associated with diabetic complications [34]. Females were more likely to have DPN and DR than males were, consistent with previous studies [35]. We found that the association between microvascular complication and onset of T1D peaked around age 20. This is slightly older than the reported increased microvascular complication risk with T1D onset around puberty [11], but this may reflect a skew present in our cross-sectional cohort. Contrary to previous reports, we did not find any association between total cholesterol and diabetic complication risk [36]. This difference in association between these cohorts is potentially due to the different populations studied.

We showed that the presence of one diabetic complication is strongly associated with having other diabetic complications (Table 2). This observation suggests that similar clinical variables may be used to predict multiple diabetic complications. Our models support this hypothesis since a logistic regression model including age, age at T1D diagnosis, average SBP, and average HbA1c performed well (Figure 1) with optimism-corrected R2 and Harrell’s C statistics 0.39 and 0.87 for DPN, 0.24 and 0.86 for AN, 0.49 and 0.91 for DR, and 0.22 and 0.83 for DN, respectively. This compares favorably to previously reported models [16,17,18,19,20] in terms of discrimination and calibration with the advantage of improved model simplicity and interpretability.

While other variables have been associated with T1D complications and applied in other risk models, we wanted to minimize the number of variables patients or clinicians need to identify to calculate complication risks. Namely, we did not include body mass index, triglycerides, or diastolic blood pressure [34]. Model simplicity might sacrifice the model’s discriminatory and calibration properties, but we have demonstrated that our models are comparable to previously reported models.

One of the limitations of this study is its cross-sectional nature. This does not allow for temporal analyses which can help to establish causation; for example, we are not able to discern if use of a statin or angiotensin-converting enzyme inhibitor is associated with complications directly or via the diagnosis leading to use of the medication. The cross-sectional design also means we were unable to update our data with changes in diagnoses of complications, so our study likely underestimates the rates of diabetic microvascular complications. While we were able to address our models’ accuracy and calibration, validation is still required to establish generalizability. A third limitation due to the cross-sectional design is that the management of T1D including medications and screening for complications has invariably changed since patient recruitment ended in 2010, and we are not able to account for possible effects this might have had in predicting T1D complications.

Outside the above limitations owing to the cross-sectional design, the study population was entirely Caucasian. Although non-Hispanic Caucasians have the highest rate of T1D in the U.S., an estimated 23% of T1D is outside this demographic [37,38]. Thus, it is possible that the results of our models are not generalizable to the entire T1D population. An additional limitation to generalizability is that we have not yet externally validated our models in an independent population, only via bootstrapping. Since the microvascular complication data were obtained from an electronic health record and not through regular screening, we would expect the complications to be underdiagnosed. Indeed, our calibration plots do all indicate the models predict a higher rate of diabetic complications compared with the training data.

We emphasize the important role of the modifiable factors HbA1c and SBP in the risk of developing microvascular complications (Figure 2) and of informing patients of the potential reductions in risk associated with decreases in HbA1c and SBP. We hope this risk calculator becomes a useful tool for clinicians and patients and helps motivate patients to modify their risk factors for diabetic complications (https://ptran25.shinyapps.io/Diabetic_Peripheral_Neuropathy_Risk, (accessed on 14 October 2021)). The risk calculator is not intended to replace or support clinician diagnosis of microvascular complications. It is merely a tool to augment lifestyle counseling.

## 5. Conclusions

We characterized demographics, past medical history, blood pressure, and laboratory values associated with four diabetic microvascular complications: DPN, AN, DR, and DN. We developed a clinical risk score for each microvascular complication using four clinical variables: age, age at T1D diagnosis, average SBP, and average HbA1C. The retinopathy model also included duration of T1D. We implemented this application as a web interface for clinicians and patients to easily calculate their risks of diabetic microvascular complications.

## Figures and Tables

**Table 1:** Demographics and clinical data of Caucasian subjects with type 1 diabetes (*n* = 1647) enrolled in PAGODA study.

Demographic/Clinical Variable	T1D (*n* = 1026)	T1D_wComp * (*n* = 621)	*p*
Male (*n* (%))	499 (48.6%)	291 (46.9%)	
Females (*n* (%))	527 (51.4%)	330 (53.1%)	n.s
Age (Years, median (range))	16.2 (12.0–25.7)	48.2 (38.9–58.47)	<0.0001
Duration of T1D (Years, median (range))	8.6 (2.1–12.5)	25.3 (14.6–34.8)	<0.0001
**Complications, *n* (%)**			
DPN		199 (32.0%)	<0.0001
AN		63 (10.1%)	<0.0001
DR		244 (39.3%)	<0.0001
DN		88 (14.2%)	<0.0001
Photocoagulation		167 (26.9%)	<0.0001
Blindness		42 (6.8%)	<0.0001
Diabetic Foot Ulcer		25 (4.0%)	<0.0001
Amputation		17 (2.7%)	<0.0001
**Past Medical History, *n* (%)**			
Hypertension		287 (46.2%)	<0.0001
Dyslipidemia		356 (57.3%)	<0.0001
Coronary Artery Disease		102 (16.4%)	<0.0001
Prior Myocardial Infarction		31 (5.0%)	<0.0001
Prior Angioplasty Stent		61 (9.8%)	<0.0001
Prior CABG		44 (7.1%)	<0.0001
Prior Transient Ischemic Attack		14 (2.3%)	<0.0001
Prior Cerebrovascular Accident		9 (1.4%)	<0.0001
Smoking Status†			
Current	32 (3.1%)	73 (11.8%)	<0.0001
Former	58 (5.7%)	157 (25.3%)	
Never	898 (87.5%)	383 (61.7)	
**Physiologic Measurements and Laboratory Values, Median (IQR)**			
Systolic Blood Pressure (mmHg)	114.7 (108.6–122)	123.3 (115.7–131.7)	<0.0001
Diastolic Blood Pressure (mmHg)	70 (66.3–74.8)	72.7 (70.0–78.0)	<0.0001
Creatinine (mg/dL)	0.77 (0.6–0.9)	0.97 (0.83–1.1)	<0.0001
Albumin Creatinine Ratio (mg/ug)	9.04 (5.4–18.5)	9.91 (5.2–43.4)	<0.05
Lipid panel			
Total Cholesterol (mg/dL)	163.0 (146.0–185.3)	175.5 (150.8–200.0)	<0.0001
LDL (mg/dL)	90.0 (77.0–106.0)	96.5 (76.0–118.5)	<0.001
HDL (mg/dL)	53.0 (43.0–64.0)	55.0 (44.8–68.0)	<0.01
Triglycerides (mg/dL)	76.0 (55.0–112.0)	79.0 (58.0–119.0)	n.s.
HbA1C (NGSP, %)	7.9 (7.1–8.9)	7.8 (7.3–8.7)	n.s.
HbA1c (IFCC, mmol/mol)	63.6 (54.1–73.0)	62.1(55.2–71.2)	n.s.

* T1D patients with any complications. DPN: diabetic peripheral neuropathy, AN: autonomic neuropathy, DR: diabetic retinopathy, DN: diabetic nephropathy, † smoking status was not available for 46 subjects.

**Table 2:** Univariate association of clinical variables with diabetic complications.

	DPN		AN		DR		DN	
Demographics	OR	*p*	OR	*p*	OR	*p*	OR	*p*
Age	1.1	<0.0001	1.1	<0.0001	1.1	<0.0001	1.0	<0.0001
Age at T1D diagnosis	1.0	<0.0001	1.0	0.0015	1.0	0.0039	1.0	0.0310
Duration of T1D	1.1	<0.0001	1.1	<0.0001	1.1	<0.0001	1.1	<0.0001
Sex	0.7	0.0150	1.1	0.6500	0.7	0.0038	0.9	0.4800
**Complications**								
DPN			25.4	<0.0001	12.2	<0.0001	7.9	<0.0001
AN	25.4	<0.0001			9.3	<0.0001	6.0	<0.0001
DR	12.2	<0.0001	9.3	<0.0001			13.7	<0.0001
DN	7.9	<0.0001	6.0	<0.0001	13.7	<0.0001		
Blindness	6.6	<0.0001	5.6	<0.0001	286.0	<0.0001	19.1	<0.0001
Photocoagulation	10.5	<0.0001	8.1	<0.0001	1492.5	<0.0001	14.5	<0.0001
Amputation	36.2	<0.0001	11.4	<0.0001	19.6	<0.0001	6.1	0.0021
Diabetic Foot Ulcer	198.3	<0.0001	16.8	<0.0001	32.8	<0.0001	14.1	<0.0001
**Past Medical History**								
Smoking	1.5	0.1400	1.0	0.9200	1.5	0.1200	1.4	0.4000
Hypertension	6.8	<0.0001	4.9	<0.0001	7.9	<0.0001	8.1	<0.0001
Dyslipidemia	5.0	<0.0001	4.6	<0.0001	3.7	<0.0001	3.3	<0.0001
CAD	13.3	<0.0001	6.1	<0.0001	6.0	<0.0001	5.8	<0.0001
Prior Angioplasty/Stent	18.5	<0.0001	6.1	<0.0001	8.9	<0.0001	7.4	<0.0001
Prior CABG	12.0	<0.0001	5.4	0.0001	5.6	<0.0001	4.2	0.0004
Prior CVA	9.4	0.0009	13.5	0.0003	11.8	0.0005	15.0	<0.0001
Prior MI	12.7	<0.0001	4.0	0.0120	7.5	<0.0001	5.5	0.0001
Prior TIA	19.5	<0.0001	4.5	0.0540	4.5	0.0059	14.4	<0.0001
**Physiologic Measurements and Laboratory Values**								
SBP	1.1	<0.0001	1.0	0.0001	1.1	<0.0001	1.1	<0.0001
DBP	1.0	0.0021	1.1	0.0005	1.0	0.0002	1.1	0.0007
Hemoglobin	0.8	<0.0001	0.8	0.0600	0.7	<0.0001	0.7	<0.0001
Albumin	1.0	0.3200	0.7	0.3800	0.3	<0.0001	0.4	0.0011
BUN	1.1	<0.0001	1.0	0.0087	1.1	<0.0001	1.1	<0.0001
Creatinine	1.0	0.6700	1.0	0.9100	1.0	0.4500	1.1	0.0200
Micro Albumin	1.0	0.0084	1.0	0.8400	1.0	<0.0001	1.0	<0.0001
ACR	1.0	0.0220	1.0	0.8000	1.0	<0.0001	1.0	<0.0001
Lipid panel								
Total Cholesterol	1.0	0.1	1.0	0.5	1.0	0.0290	1.0	0.1
LDL	1.0	0.7	1.0	0.6	1.0	0.3	1.0	0.8
HDL	1.0	0.02	1.0	0.0027	1.0	0.02	1.0	0.8
Triglycerides	1.0	0.4	1.0	0.3	1.0	0.8	1.0	0.1
HbA1c	1.0	0.8	1.0	0.8	0.9	0.2	1.1	0.4
HbA1c, SD	0.9	0.3	0.8	0.4	0.8	0.0	1.0	0.8
HbA1c, last visit	1.0	0.8	1.0	1.0	0.9	0.1	1.1	0.4
HbA1c, maximum	1.0	1.0	1.0	0.9	0.9	0.1	1.1	0.2

DPN: diabetic peripheral neuropathy, AN: autonomic neuropathy, DR: diabetic retinopathy, DN: diabetic nephropathy, SD: standard deviation, ACR: albumin–creatinine ratio, CAD: coronary artery disease, CABG: coronary artery bypass graft, CVA: Cerebrovascular accident, MI: myocardial infarction, TIA: transient ischemic attack, DBP: diastolic blood pressure, SBP: systolic blood pressure.

## Data Availability

Code and data used in this paper are available in github repository at https://github.com/pmtran5884/T1D_Complications (accessed on 14 October 2021).

## Supplementary Materials

The following are available online at https://www.mdpi.com/article/10.3390/ijerph182111094/s1, Figure S1: Calibration plots and nomograms for DPN (A & B, AN (C & D), DR (E & F), and DN (G & H), Table S1: Univariate association of clinical variables with diabetic complications.
