# Supplementary material for: T1DMicro: A Clinical Risk Calculator for Type 1 Diabetes Related Microvascular Complications
Source: Int J Environ Res Public Health. 2021 Oct 21;18(21):11094. doi: 10.3390/ijerph182111094 (PMC8583376; doi:10.3390/ijerph182111094)
Supplement: Supplementary file 1 [file ijerph-18-11094-s001.zip › ijerph-1385687-supplementary.pdf]

Supplementary Table S1. Univariate association of clinical variables with diabetic complications

	DPN		CAN		DR		DN	
	OR (95%CI)	p-value	OR (95%CI)	p-value	OR (95%CI)	p-value	OR (95%CI)	p-value
Age	1.1 (1.1-1.1)	2.49E-49	1.1 (1-1.1)	6.73E-15	1.1 (1.1-1.1)	5.05E-50	1 (1-1.1)	2.81E-16
Age at T1D diagnosis	1 (1-1.1)	9.81E-22	1 (1-1)	1.46E-03	1 (1-1)	3.85E-03	1 (1-1)	3.09E-02
Duration of T1D	1.1 (1.1-1.1)	1.28E-45	1.1 (1.1-1.1)	2.21E-16	1.1 (1.1-1.1)	2.26E-65	1.1 (1.1-1.1)	7.77E-21
Sex	0.7 (0.5-0.9)	1.46E-02	1.1 (0.7-1.9)	6.46E-01	0.7 (0.5-0.9)	3.77E-03	0.9 (0.6-1.3)	4.83E-01
**Complications**								
DPN			25.4 (14.2-45.5)	1.55E-27	12.2 (8.8-17)	4.77E-49	7.9 (5-12.4)	2.79E-19
AN	25.4 (14.2-45.5)	1.55E-27			9.3 (5.5-15.7)	8.97E-17	6 (3.2-11.4)	4.26E-08
DR	12.2 (8.8-17)	4.77E-49	9.3 (5.5-15.7)	8.94E-17			13.7 (8.6-21.7)	2.15E-28
DN	7.9 (5-12.4)	2.79E-19	6 (3.2-11.4)	4.26E-08	13.7 (8.6-21.7)	2.16E-28		
Blindness	6.6 (3.5-12.4)	3.39E-09	5.6 (2.4-13.1)	8.08E-05	286 (39.1-2090.5)	2.51E-08	19.1 (9.9-36.9)	1.92E-18
Photocoagulation	10.5 (7.4-15.1)	6.57E-38	8.1 (4.7-13.7)	1.43E-14	1492.5 (363.3-6131.9)	3.79E-24	14.5 (9.2-23)	3.80E-30
Amputation	36.2 (10.3-127.2)	2.14E-08	11.4 (3.9-33.5)	9.26E-06	19.6 (6.3-60.6)	2.41E-07	6.1 (1.9-19.3)	2.10E-03
Diabetic Foot Ulcer	198.3 (26.7-1474.5)	2.36E-07	16.8 (7.1-39.9)	1.36E-10	32.8 (11.1-96.4)	2.28E-10	14.1 (6.1-32.7)	7.91E-10
**Past Medical History**								
Smoking	1.5 (0.9-2.7)	1.44E-01	1 (0.4-2.3)	9.18E-01	1.5 (0.9-2.7)	1.21E-01	1.4 (0.6-3)	3.96E-01
Hypertension	6.8 (5-9.4)	3.40E-32	4.9 (2.9-8.2)	1.55E-09	7.9 (5.8-10.6)	1.99E-41	8.1 (5.2-12.7)	4.63E-20
Dyslipidemia	5 (3.7-6.8)	1.86E-24	4.6 (2.8-7.7)	4.60E-09	3.7 (2.8-5)	3.24E-19	3.3 (2.1-5.1)	8.02E-08
CAD	13.3 (8.7-20.5)	4.61E-32	6.1 (3.3-11.2)	6.33E-09	6 (3.9-9.1)	5.29E-17	5.8 (3.4-9.9)	2.05E-10
Prior Angioplasty Stent	18.5 (10.6-32.4)	1.52E-24	6.1 (2.9-12.7)	1.45E-06	8.9 (5.3-15.1)	4.66E-16	7.4 (4-13.8)	1.99E-10
Prior CABG	12 (6.4-22.3)	4.63E-15	5.4 (2.3-12.6)	1.09E-04	5.6 (3.1-10.3)	2.64E-08	4.2 (1.9-9.3)	4.24E-04
Prior CVA	9.4 (2.5-35.2)	9.20E-04	13.5 (3.3-55.2)	3.03E-04	11.8 (2.9-47.7)	5.06E-04	15 (4-57)	6.78E-05
Prior MI	12.7 (6.1-26.5)	1.66E-11	4 (1.4-11.9)	1.19E-02	7.5 (3.6-15.3)	4.75E-08	5.5 (2.3-13.1)	1.29E-04
Prior TIA	19.5 (6.1-62.9)	6.35E-07	4.5 (1-20.4)	5.38E-02	4.5 (1.5-13)	5.95E-03	14.4 (4.9-42.4)	1.38E-06
**Physiologic Measurements and Laboratory Values**								
SBP	1.1 (1-1.1)	5.97E-15	1 (1-1.1)	1.18E-04	1.1 (1-1.1)	3.07E-19	1.1 (1-1.1)	2.02E-10
DBP	1 (1-1.1)	2.07E-03	1.1 (1-1.1)	5.20E-04	1 (1-1.1)	1.66E-04	1.1 (1-1.1)	6.89E-04
Hemoglobin	0.8 (0.7-0.8)	3.46E-06	0.8 (0.7-1)	6.00E-02	0.7 (0.6-0.8)	6.06E-08	0.7 (0.6-0.8)	5.39E-05
Albumin	1 (1-1)	3.19E-01	0.7 (0.4-1.5)	3.75E-01	0.3 (0.2-0.5)	3.73E-07	0.4 (0.2-0.7)	1.15E-03
BUN	1.1 (1.1-1.1)	8.08E-12	1 (1-1.1)	8.66E-03	1.1 (1.1-1.2)	1.18E-19	1.1 (1.1-1.2)	3.06E-17
Creatnine	1 (1-1.1)	6.72E-01	1 (0.9-1.1)	9.06E-01	1 (1-1.1)	4.48E-01	1.1 (1-1.1)	1.99E-02
Micro-Albumin	1 (1-1)	8.42E-03	1 (1-1)	8.42E-01	1 (1-1)	9.42E-05	1 (1-1)	9.71E-06
ACR	1 (1-1)	2.21E-02	1 (1-1)	8.00E-01	1 (1-1)	1.54E-06	1 (1-1)	3.00E-10
Lipid Panel								
LDL	1 (1-1)	7.01E-01	1 (1-1)	5.71E-01	1 (1-1)	3.28E-01	1 (1-1)	7.90E-01
Total_Cholesterol	1 (1-1)	1.15E-01	1 (1-1)	5.35E-01	1 (1-1)	2.92E-02	1 (1-1)	1.18E-01
Triglycerides	1 (1-1)	4.37E-01	1 (1-1)	2.56E-01	1 (1-1)	7.89E-01	1 (1-1)	9.33E-02
HDL	1 (1-1)	1.80E-02	1 (1-1)	2.69E-03	1 (1-1)	1.62E-02	1 (1-1)	7.55E-01
HbA1c_3.1	1 (0.9-1.1)	8.33E-01	1 (0.9-1.2)	7.52E-01	0.9 (0.8-1)	1.89E-01	1.1 (0.9-1.2)	4.21E-01
HbA1c, SD	0.9 (0.7-1.1)	3.38E-01	0.8 (0.5-1.3)	3.54E-01	0.8 (0.6-1)	4.46E-02	1 (0.8-1.3)	8.28E-01
HbA1c, last visit	1 (0.9-1.1)	7.55E-01	1 (0.8-1.2)	9.71E-01	0.9 (0.8-1)	1.26E-01	1.1 (0.9-1.2)	3.59E-01
HbA1c, maximum	1 (0.9-1.1)	9.57E-01	1 (0.8-1.1)	8.58E-01	0.9 (0.9-1)	8.40E-02	1.1 (1-1.2)	1.96E-01

DPN: Diabetic Peripheral Neuropathy, AN: Autonomic Neuropathy, DR: Diabetic Retinopathy, DN: Diabetic Nephropathy, SD: Standard Deviation, ACR: Albumin-Creatinine Ratio, CAD: Coronary Artery Disease, CABG: Coronary Artery Bypass Graft, CVA: Cerebro-vascular accident, MI: Myocardial Infarction, TIA: Transient Ischemic Attack, DBP: Diastolic Blood pressure, SBP: Systolic Blood Pressure.

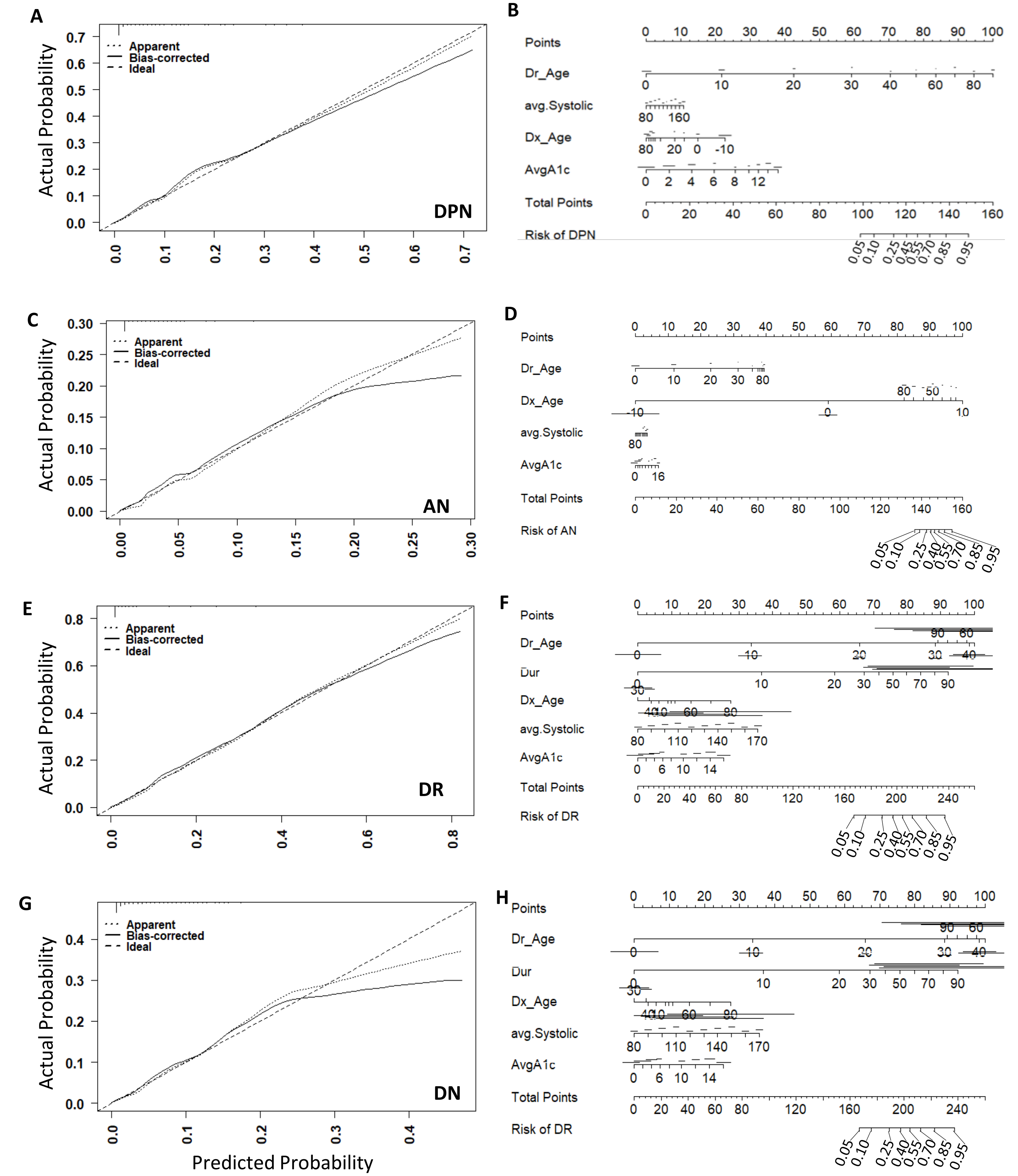

Supplementary Figure 1-X.S : Calibration plots and nomograms for DPN (A & B, AN (C & D), DR (E &F), and DN (G & H) are presented in supplementary figure 1. We applied the four microvascular complication models to the PAGODA dataset with 500 iterations of bootstrapping to generate the calibration plot showing the actual probability of complication in the y-axis and the predicted probability of complication based on the models in the x-axis. A bias correction was applied by calculating the difference in probability between the bootstrap iterations and the model prediction with the full dataset. The nomogram was generated again based on the beta coefficients from the four logistic microvascular complication models.
